# Supplementary material for: Fine Mapping and Identification of a Candidate Gene of Downy Mildew Resistance, RPF2, in Spinach (Spinacia oleracea L.)
Source: Int J Mol Sci. 2022 Nov 28;23(23):14872. doi: 10.3390/ijms232314872 (PMC9737595; doi:10.3390/ijms232314872)
Supplement: Supplementary file 1 [file ijms-23-14872-s001.zip › ijms-2022939-supplementary.pdf]

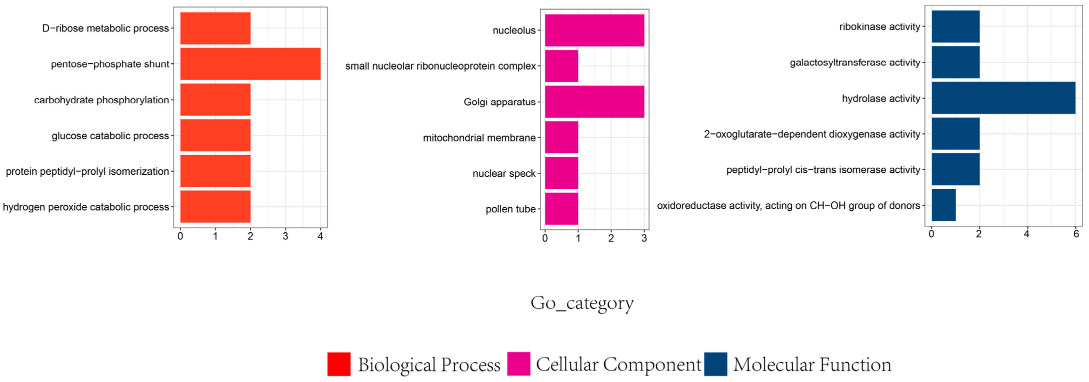

Supplementary Figure S1. The annotation of 76 genes in the 1.11Mb to 1.72Mb on chromosome 3

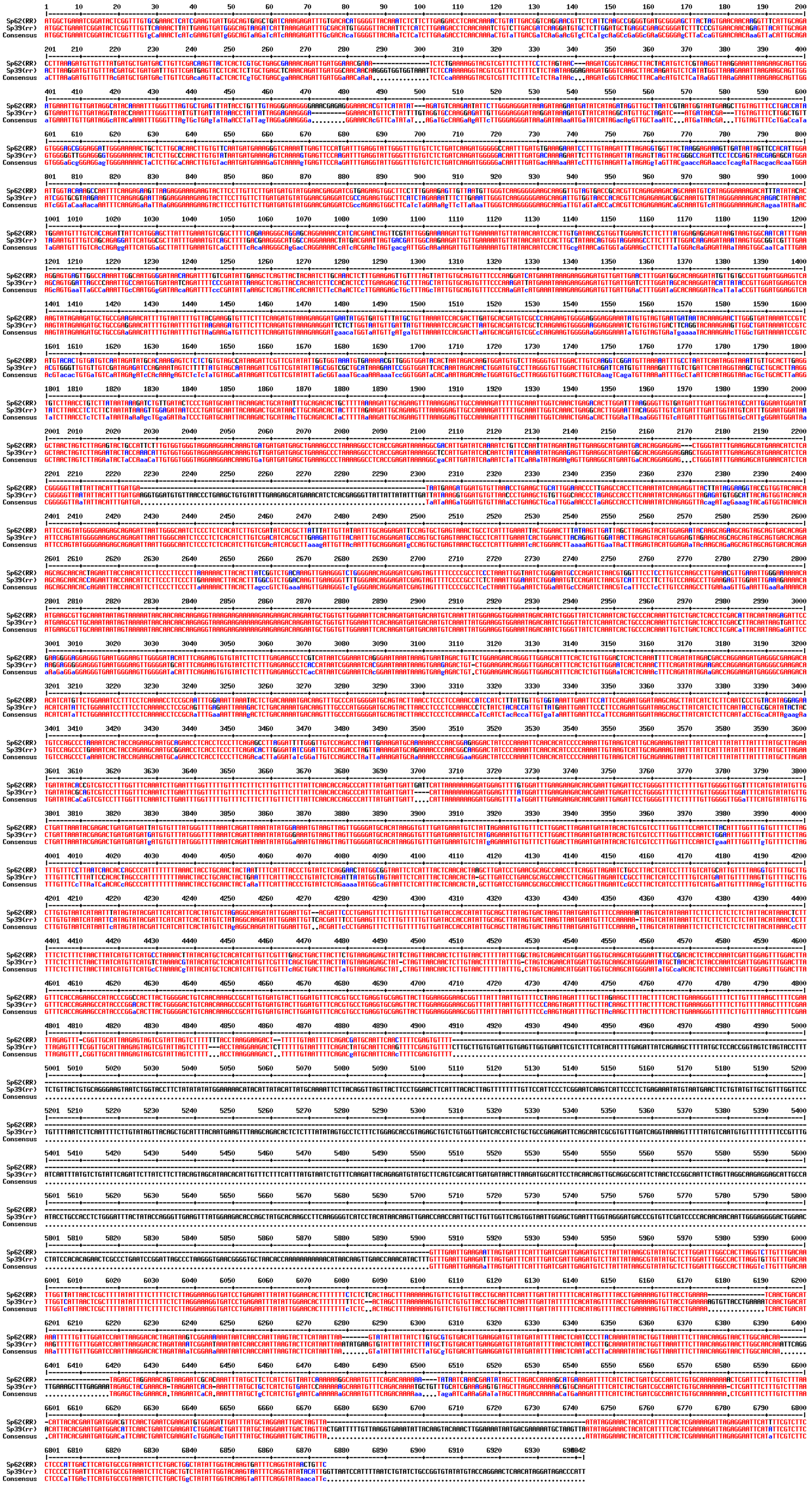

Supplementary Figure S2. The result of *Spo12821* DNA sequence in Sp39(rr) and Sp62(RR)
